# Portable Paper-Based Colorimetric Biosensor for Rapid Screening of Organophosphate Exposure via Acetylcholinesterase Activity and RGB Image Analysis

**DOI:** 10.3390/bios16090490

**Published:** 2026-09-03

**Authors:** Carlos E. Zambra, Jorge Morales-Ferreiro, Francisca Herrera Vielma, Jessica Zúñiga-Hernández

**Affiliations:** 1 Department of Industrial Technologies, Faculty of Engineering, University of Talca, Curicó 3640000, Chile; czambra@utalca.cl; 2 School of Science, Engineering and Technology, School of Engineering, Universidad Mayor, Santiago 7500994, Chile; jorge.morales@umayor.cl; 3 Center for Applied Nanotechnology (CNAP), School of Science, Engineering and Technology, Universidad Mayor, Santiago 7500994, Chile; 4 Pharmacology Laboratory, Biomedical Sciences Department, Faculty of Health Sciences, University of Talca, Talca 3465548, Chile; f.herrera.vielma@gmail.com

**Keywords:** organophosphate exposure, paper-based device, colorimetric detection, point-of-care testing

## Abstract

Background: Rapid screening of acetylcholinesterase (AChE) inhibition is essential for monitoring exposure to organophosphate compounds, particularly in field settings where access to laboratory infrastructure is limited. This study aimed to develop a portable paper-based colorimetric biosensor for the semi-quantitative detection of AChE activity in blood samples. Methods: The biosensor was based on a pH-dependent color change adapted from the modified Edson method. The platform was first optimized using experimental models and then evaluated in human capillary blood samples collected under real field conditions. Reference serum cholinesterase activity was determined by a certified clinical laboratory and used for comparison with the colorimetric response of the biosensor. RGB (red, green, and blue) image analysis was performed in a subset of samples to digitally characterize the chromatic response of the device. Results: Samples with preserved AChE activity showed a visible color shift associated with substrate hydrolysis, whereas samples with reduced or inhibited activity maintained darker blue-dominant tones. RGB analysis of human samples revealed significant associations between AChE activity and the R and G channels, supporting the ability of the platform to distinguish between chromatic patterns associated with different ranges of enzymatic activity. Conclusions: The proposed platform demonstrated the feasibility of translating a pH-based AChE assay into a portable paper-based format for semi-quantitative visual and RGB-assisted screening. Its evaluation in human samples demonstrated the feasibility of its use under field conditions as a proof-of-concept. Further studies are needed to optimize its analytical performance and validate its application in larger and more diverse populations.

## 1. Introduction

Agriculture is a key productive activity worldwide and relies heavily on the use of pesticides to increase crop yield and quality and to ensure food security for a growing global population [1]. Among these, organophosphate (OP) compounds are widely used because of their high insecticidal efficacy, although their application has been associated with adverse effects on human health and the environment [2]. In South America, where agriculture represents a major economic sector, intensive and often prolonged OP use has raised concerns regarding occupational and environmental exposure, particularly in rural communities [3]. In Chile, pesticide use is widespread and constitutes an important strategy for improving crop productivity and quality [4]. However, prolonged and inappropriate use of OP compounds has raised concerns due to their environmental persistence and potential bioaccumulation, especially in agricultural regions such as Maule, where intensive fruit production represents one of the main economic activities [5]. Exposure to these compounds, either through direct contact or environmental contamination, has been associated with neurological, endocrine, and reproductive disorders, as well as an increased risk of chronic diseases [6]. These conditions have raised concerns regarding environmental persistence and bioaccumulation in exposed organisms [7]. These conditions highlight the importance of implementing reliable strategies for monitoring OP exposure under real working conditions.

Conventional analytical methods for detecting OP residues in biological or environmental samples are highly sensitive and are mainly based on chromatographic techniques such as gas or liquid chromatography coupled to mass spectrometry; however, they require specialized equipment, centralized laboratories, and lengthy analysis [8,9]. This reality limits their applicability for field surveillance in agricultural communities and for the implementation of early warning systems. Consequently, simpler, faster, and lower-cost methodologies have been explored, including colorimetric methods based on AChE activity, a key enzymatic target of OP toxicity. OP exposure leads to AChE inhibition, resulting in the accumulation of acetylcholine and subsequent neurotoxic effects, which can be indirectly detected through enzyme-based colorimetric reactions that generate a visible color change. These tests offer advantages such as portability, low infrastructure requirements, and rapid interpretation of results, making them attractive tools for community-based and occupational monitoring of OP exposure [10,11,12].

In recent years, optical approaches have become attractive alternatives for AChE inhibition screening because they combine rapid analysis with simple operation and portability. In particular, paper-based colorimetric devices integrated with digital image analysis have improved the feasibility of objective field-based screening while reducing the need for specialized laboratory infrastructure [13,14,15,16,17,18].

The present study builds upon Edson’s colorimetric method, later modified by Limperos and Ranta [10], which is based on the hydrolysis of acetylcholine by AChE, generating acetic acid and causing a decrease in pH that can be detected using acid–base indicators. Inhibition of this reaction by OP compounds results in reduced acidification and, consequently, altered color development, allowing indirect assessment of AChE activity [11]. In this work, the original laboratory-based protocol was adapted and optimized for implementation in a portable test strip format.

The aim of this study was to develop and evaluate a portable paper-based colorimetric biosensor by adapting the classical Edson–Limperos assay for rapid field screening of AChE inhibition in finger-prick capillary whole blood, incorporating RGB image analysis as a complementary tool for objective color evaluation. This approach combines low-cost fabrication, visual colorimetric detection, and digital image analysis into a simple screening tool suitable for point-of-care and field applications. Furthermore, the device was evaluated using human capillary blood samples collected under real-world conditions, supporting its potential applicability for environmental and occupational monitoring of OP exposure.

## 2. Materials and Methods

### 2.1. Ethics Statement for Animal Experiments

The protocols and procedures with experimental animals (male Sprague–Dawley rats, body weight 250–300 g) were conducted in accordance with the Guide for the Care and Use of Laboratory Animals, 8th edition (National Academies Press, Washington, DC, USA, 2011), and were approved by the Bioethics Committee for Care and Research in Animals of the University of Talca (Approval Folio: 2021-04-A). All animal procedures were performed under the supervision of an experienced veterinarian.

### 2.2. Ethics Statement for Human Participants

This study was approved by the Re-accredited Scientific Ethics Committee of the University of Talca, accredited by the Regional Ministerial Health Secretariat of Maule (SEREMI de Salud del Maule), in accordance with Resolution Exenta No. 1976, dated 28 September 2023. Authorization was also obtained from the Municipal Health Departments of the municipalities of Colbún and San Rafael.

### 2.3. Reagents and Experimental Treatments

Diazinon (O,O-diethyl O-[6-methyl-2-(1-methylethyl)-4-pyrimidinyl] phosphorothioate) was used as an AChE inhibitor compound. The pesticide was administered to Sprague–Dawley rats via intraperitoneal injection at a dose of 10 mg/kg body weight. Blood samples were collected from the same animals immediately before diazinon administration (0 min) and again 30 min after administration to evaluate AChE activity using the colorimetric method described above.

### 2.4. Colorimetric Reaction Principle

The Edson method, as modified by Limperos and Ranta [10], was adapted to establish the colorimetric sensing principle of the proposed biosensor. Initial optimization experiments were performed using serum samples under controlled laboratory conditions to optimize the reaction conditions. Subsequently, the optimized assay was applied to whole-blood samples obtained from human participants and Sprague–Dawley rats. Initially, both acetylcholine iodide (AChI; C_7_H_16_INO_2_) and AChClO_4_ were evaluated as substrates. For the colorimetric reaction, 10 µL of whole blood or serum was mixed with 500 µL of prepared reagents, consisting of bromothymol blue (BTB, 0.0448%, pH 7.2) and 500 µL of substrate solution (10 µM AChI or AChClO_4_), in Eppendorf tubes. The reaction mixtures were incubated for 20 min at room temperature, after which the color change was recorded following the original protocol. Appropriate controls and standards were included as specified in the method. Since both substrates generated comparable responses and AChClO_4_ provided a more easily distinguishable visual color transition, AChClO_4_ was selected for all subsequent experiments.

### 2.5. Modification of the Edson Method for Microvolume Testing in 96-Well ELISA Plates

To facilitate assay miniaturization and the subsequent development of the paper-based device, the method was adapted to microvolume conditions. Briefly, 10 μL of serum were mixed with 30 μL of BTB solution (0.0448%, pH 7.2) and 30 μL of AChClO_4_ (10 μM) as substrate. The mixtures were incubated in 96-well ELISA plates at room temperature, and color changes were visually evaluated after 20 min. Positive and negative controls were included according to the original Edson protocol to ensure reaction specificity and exclude non-enzymatic interference. For this, blank controls correspond to all reagents in the absence of biological sample, while no-substrate control corresponds to all reagents without AChClO_4_.

### 2.6. Test Strip Fabrication

Test strips measuring 0.5 cm × 3 cm were prepared from different types of backing paper, including Whatman LF1 paper, chromatography paper, nitrocellulose paper, and filter paper, in order to evaluate their fluid retention and migration capacity. Each strip was separately impregnated with AChClO_4_, used as the enzymatic substrate, at a final concentration of 10 µM, followed by incubation at room temperature. Subsequently, the strips were dried in a laboratory oven at 50 °C until complete solvent evaporation. Next, 10 µL of whole blood from Sprague–Dawley rats was applied to one end of the strip, allowing the serum to migrate toward the central area by capillary action. Once migration was stabilized, 30 µL of BTB was added at the opposite end of the strip relative to sample application, allowing it to migrate toward the reaction zone, where interaction with the serum resulted in a visible color change. The color change was observed after 10 min of incubation at room temperature, and the result was recorded visually. The results were evaluated by comparing the intensity and homogeneity of the color change between the different types of support paper in order to identify the material with the best performance for the development of the test strips.

### 2.7. Human Participants

To validate the proposed method, field sampling was carried out in the municipalities of San Rafael and Curicó (Maule Region, Chile). After optimization of the colorimetric reaction and the paper-based platform, the final biosensor configuration was designed for direct analysis of finger-prick capillary whole blood using pre-sealed paper strips, which represents the intended field-use protocol of the device. Human capillary blood samples were collected by finger prick from a total of 68 participants and analyzed in situ using pre-sealed test strips. Each sample was applied directly to the reaction zone of the strip, allowing the reaction to occur under controlled environmental conditions. Serum cholinesterase activity was determined by a certified clinical laboratory (Laboratory Loncomilla^®^, Talca, Maule, Chile) and used as the reference measurement. The reference assay was performed on serum samples using the Roche Cholinesterase Gen.2 method, a spectrophotometric colorimetric assay for the quantitative determination of serum cholinesterase activity. Results were reported in U/L. According to the manufacturer’s instructions, the reference interval for adults was 5320–12,920 U/L. Participants were classified according to the serum cholinesterase activity measured by the reference laboratory: <7000 U/L, 7000–10,000 U/L, and >10,000 U/L. These ranges were used solely to facilitate comparison of the biosensor response across different levels of enzyme activity within the study population and should not be interpreted as established clinical thresholds for OP exposure or AChE deficiency. Due to field conditions, not all samples were processed using all analytical approaches. RGB-based image analysis was performed on a subset of samples (n = 35) selected based on image quality criteria to ensure reliable quantification. For RGB analysis, strip images were acquired under standardized conditions and processed using ImageJ software (ImageJ V 1.54k). A region of interest (ROI) was defined within the reaction zone, and mean intensity values for the red (R), green (G), and blue (B) channels were extracted for each sample. Images were included only when they showed adequate illumination, complete visualization of the reaction area, and were free of reflections or other artifacts that could interfere with color analysis. The selected samples included all three AChE activity categories evaluated in this study. The remaining samples were evaluated in situ to assess qualitative color changes of the test strips. Demographic, occupational, and biochemical characteristics of the study population are reported in Supplementary Table S1.

### 2.8. Digital RGB Analysis

A colorimetric analysis device based on the RGB method was developed to objectively quantify the color changes of the paper test strips. The strips were placed on a custom-designed 3D-printed holder and inserted into an enclosed black measurement chamber equipped with an integrated white illumination system and an upper-mounted high-resolution camera. During image acquisition, the strip remained in a fixed position under constant illumination to minimize image variability. RGB values from the reaction area were obtained using the integrated image analysis software and expressed as relative RGB intensity values. For the analysis of the human test strips, photographs were acquired using a fixed camera support under controlled illumination (Supplementary Figure S1), maintaining constant camera-to-strip distance, strip orientation, and image acquisition conditions for all photographs. The photographs were analyzed using ImageJ software (NIH, Bethesda, MD, USA). The reaction area of each LF1 paper strip was first identified, and the region of interest (ROI) was defined using a semi-automated color segmentation procedure with the Magic Select tool (Paint 3D, Microsoft V. 2024.2410.13017.0 (2024)) in Paint 3D. The operator initially identified the reaction area, after which the software automatically segmented the ROI based on differences in color and contrast. The resulting segmentation was visually reviewed and manually adjusted only when necessary to ensure that the selected ROI accurately represented the colorimetric reaction zone while excluding the whole-blood area and regions containing unreacted bromothymol blue. The final ROI was then analyzed in ImageJ to obtain the mean red (R), green (G), and blue (B) intensity values. The same segmentation procedure, ROI selection criteria, and image quality requirements were applied to all samples. A total of 68 participants were enrolled in the study; however, RGB analysis was performed on 35 samples. Images were included only if they met the minimum quality requirements for reliable RGB extraction, including adequate illumination, complete visualization of the reaction area, and the absence of reflections or artifacts that could affect color measurement. Images that did not meet these criteria were excluded from the RGB analysis.

### 2.9. Statistical Analysis

Data are presented as mean ± standard deviation (SD). Estimation plots were used for data visualization. Comparisons between two groups were performed using an unpaired Student’s *t*-test. Correlation analyses were performed using simple linear regression to evaluate the relationship between variables, and coefficients of determination (R^2^) were calculated. All analyses were performed using GraphPad Prism (11.0.0 (84), GraphPad Software, San Diego, CA, USA). Statistical significance was set at *p* < 0.05.

## 3. Results

The overall workflow of the developed device consists of four main steps: (i) application of the biological sample onto the paper substrate, (ii) capillary-driven migration of the sample through the strip, (iii) enzymatic reaction resulting in a pH dependent color change, and (iv) digital quantification of the chromatic response using RGB analysis. This design enables a rapid and portable assessment of AChE activity under both controlled and field conditions.

### 3.1. Validation of the Colorimetric Sensing Principle Based on the Modified Edson–Limperos Assay

**Figure 1:**
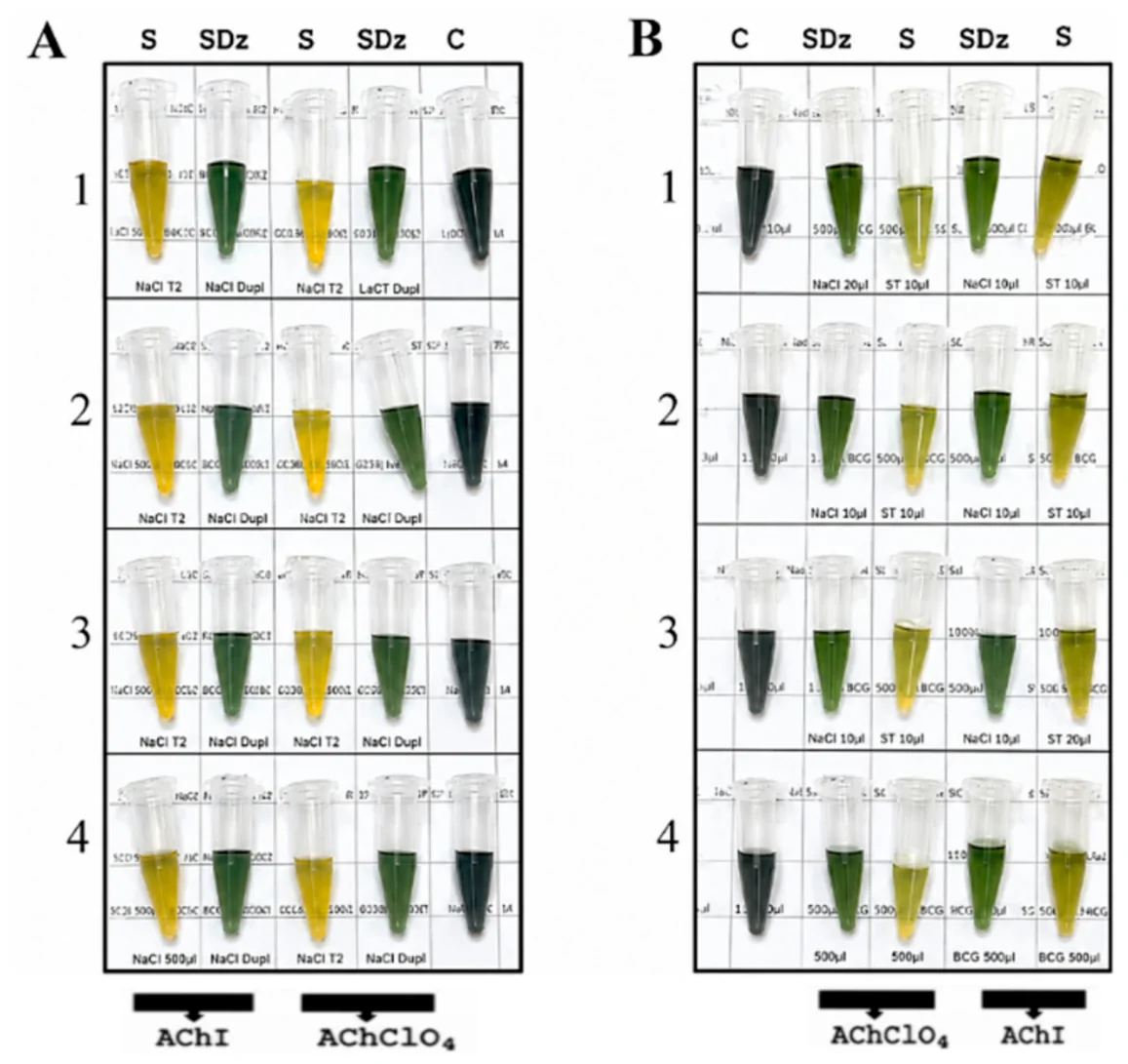
Colorimetric detection of AChE activity using the Edson method modified by Limperos and Ranta. Representative colorimetric reactions obtained from serum samples: (**A**) Sprague–Dawley rats (n = 4); (**B**) whole blood of human participants (n = 4). Rows (1–4) correspond to independent biological replicates. Columns indicate the experimental conditions. Both AChI (original method) and AChClO_4_ were evaluated, and representative reactions obtained with both substrates are shown. S, serum; SDZ, serum plus diazinon; and C, control serum.

To establish the sensing principle underlying the proposed paper-based biosensor, the colorimetric assay was first validated under controlled experimental conditions by adapting the original protocol to Eppendorf tubes and evaluating both AChI and AChClO_4_ as substrates. Since both compounds generated comparable responses and AChClO_4_ provided a more easily distinguishable visual color transition, AChClO_4_ was selected for all subsequent experiments. Under these conditions, samples with preserved AChE activity exhibited a clear chromatic shift from blue (pH 7.2) to greenish and yellow tones (pH < 5), indicating acidification of the reaction medium due to substrate hydrolysis. This response was consistently observed in Sprague–Dawley rats and in human blood samples (Figure 1A,B). In contrast, serum obtained from diazinon-treated animals and negative controls showed no detectable chromatic variation and remained blue, consistent with inhibition of AChE activity.

### 3.2. Microvolume Optimization of the Colorimetric Biosensing Platform

**Figure 2:**
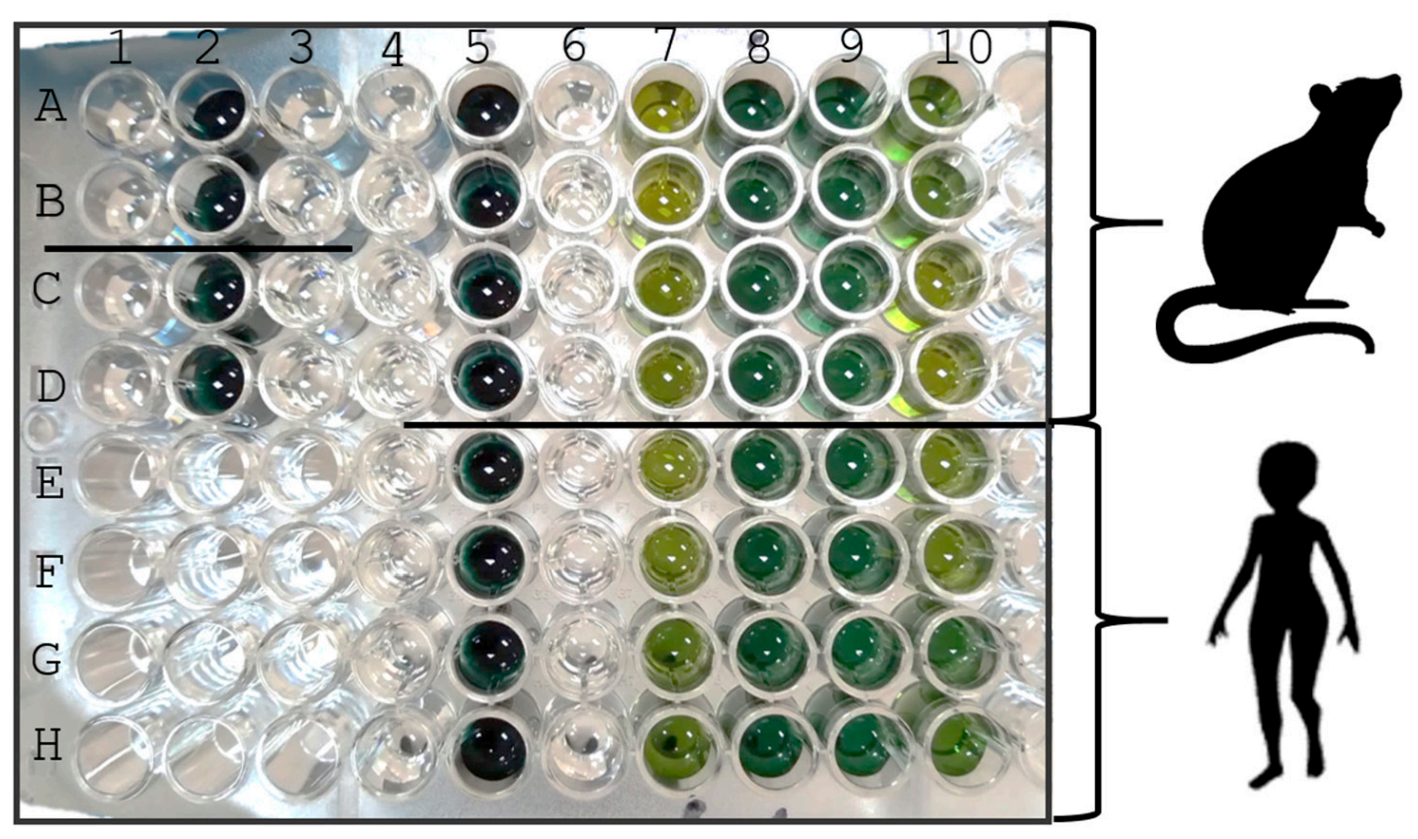
Colorimetric setup for AChE detection using the modified Edson method in microvolumes. Columns: (2) blank controls (all reagents in the absence of biological sample) used to determine the background signal. Independent reagent blanks were included for both the rat and human assays. Rows A and B correspond to the rat assay and rows C and D correspond to the human assay; (5) no-substrate controls; (7,10) reactions with AChClO_4_ (10 µM); and (8,9) reaction with AChClO_4_ (10 µM) plus diazinon. Rows correspond to independent technical replicates of (A–D) rat serum samples, and rows (E–H) human serum samples. n = 4.

To facilitate the miniaturization of the proposed biosensing platform, the modified Edson assay was adapted to reduced reaction volumes and evaluated in a 96-well ELISA plate format. The microvolume configuration yielded results consistent with those obtained using conventional Eppendorf tube assays. In mixtures containing serum with active AChE, a progressive color change from blue to greenish and yellow tones was observed after 20 min of incubation, indicating acidification of the reaction medium. This colorimetric response was similarly observed in human serum samples and Sprague–Dawley rat serum (Figure 2), demonstrating comparable enzymatic behavior between species. In contrast, samples from diazinon-treated animals and negative controls, as well as human samples lacking AChE activity, remained blue with no detectable color variation. AChClO_4_ at 10 µM was selected for all subsequent experiments, as it generated a consistent and readily distinguishable colorimetric response under reduced-volume conditions. Importantly, this miniaturization step enabled progressive reduction in the reaction volume, facilitating the development of the paper-based biosensor while preserving the ability to visually discriminate AChE activity under controlled conditions.

### 3.3. Fabrication and Optimization of the Paper-Based Biosensor

**Figure 3:**
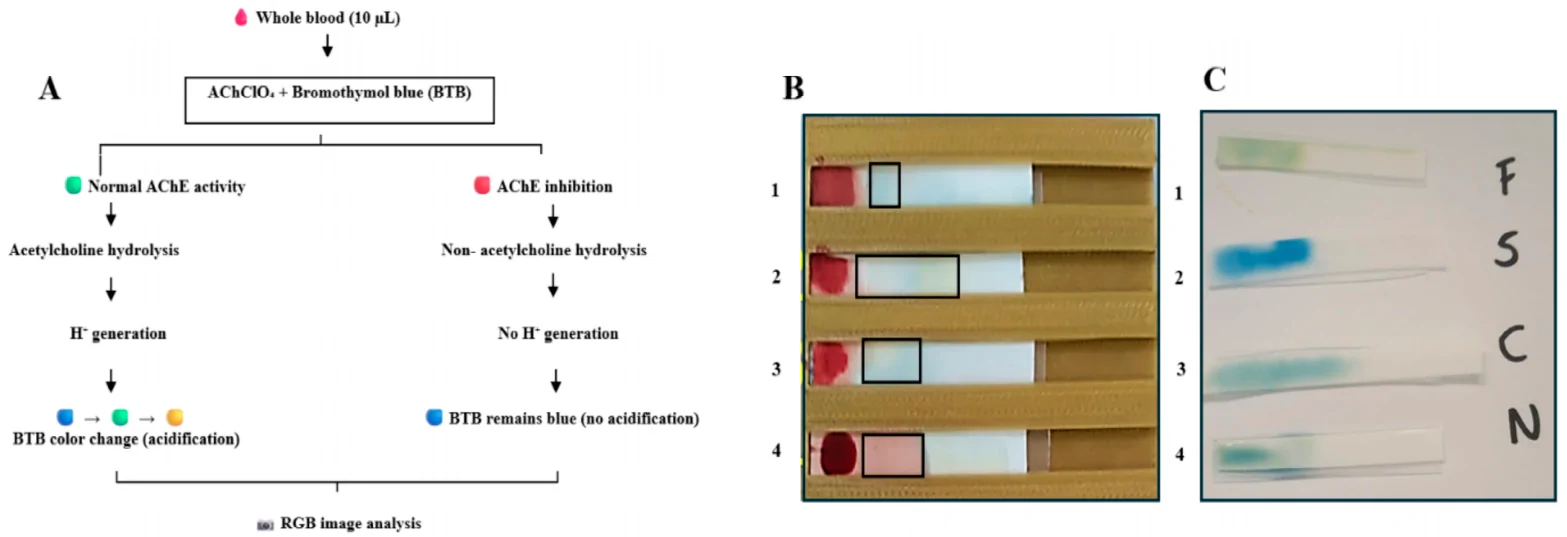
Detection principle, standardization of the support, and characterization of the paper-based biosensor. (**A**) Schematic illustration of the detection principle of the paper-based biosensor. In the presence of active AChE, acetylcholine hydrolysis generates protons that decrease the pH, promoting the color change of BTB from blue to green/yellow. When AChE activity is inhibited, hydrolysis does not occur and the indicator remains blue. (**B**) Reagent strips manufactured with different types of paper for the evaluation of capillary migration, showing representative colorimetric reactions: (1) filter paper, (2) LF1 separator paper, (3) chromatographic paper, and (4) nitrocellulose paper. (**C**) Reactive zone of the strips after reagent incorporation, corresponding to the same paper types: (1) filter paper, (2) LF1 separator paper, (3) chromatographic paper, and (4) nitrocellulose paper. All assays were performed at least in triplicate.

To optimize the proposed paper-based biosensor, different paper substrates were evaluated to identify the material providing the most suitable capillary migration while maintaining adequate fluid retention. Filter paper, Whatman LF1 paper, chromatographic paper, and nitrocellulose were assessed for this purpose. Among the tested materials, LF1 paper enabled constant and continuous serum migration, allowing clear separation and visual retention of the cellular and serum fractions, as indicated in the marked areas (Figure 3B, row 2). Following reagent deposition, the strip displayed a uniform blue coloration (Figure 3C, column 2, strip labeled “S”), consistent with the initial pH of the BTB solution (pH 7.2). These characteristics are critical for device performance, as they ensure controlled fluid transport, stable formation of the reaction zone, and reproducible colorimetric signals. Together, these findings support the suitability of the optimized strip configuration as a functional paper-based biosensor capable of integrating sample processing, reaction, and visual readout within a single platform for rapid field applications.

### 3.4. Validation of the Paper-Based Biosensor Using Human Capillary Blood Samples

**Figure 4:**
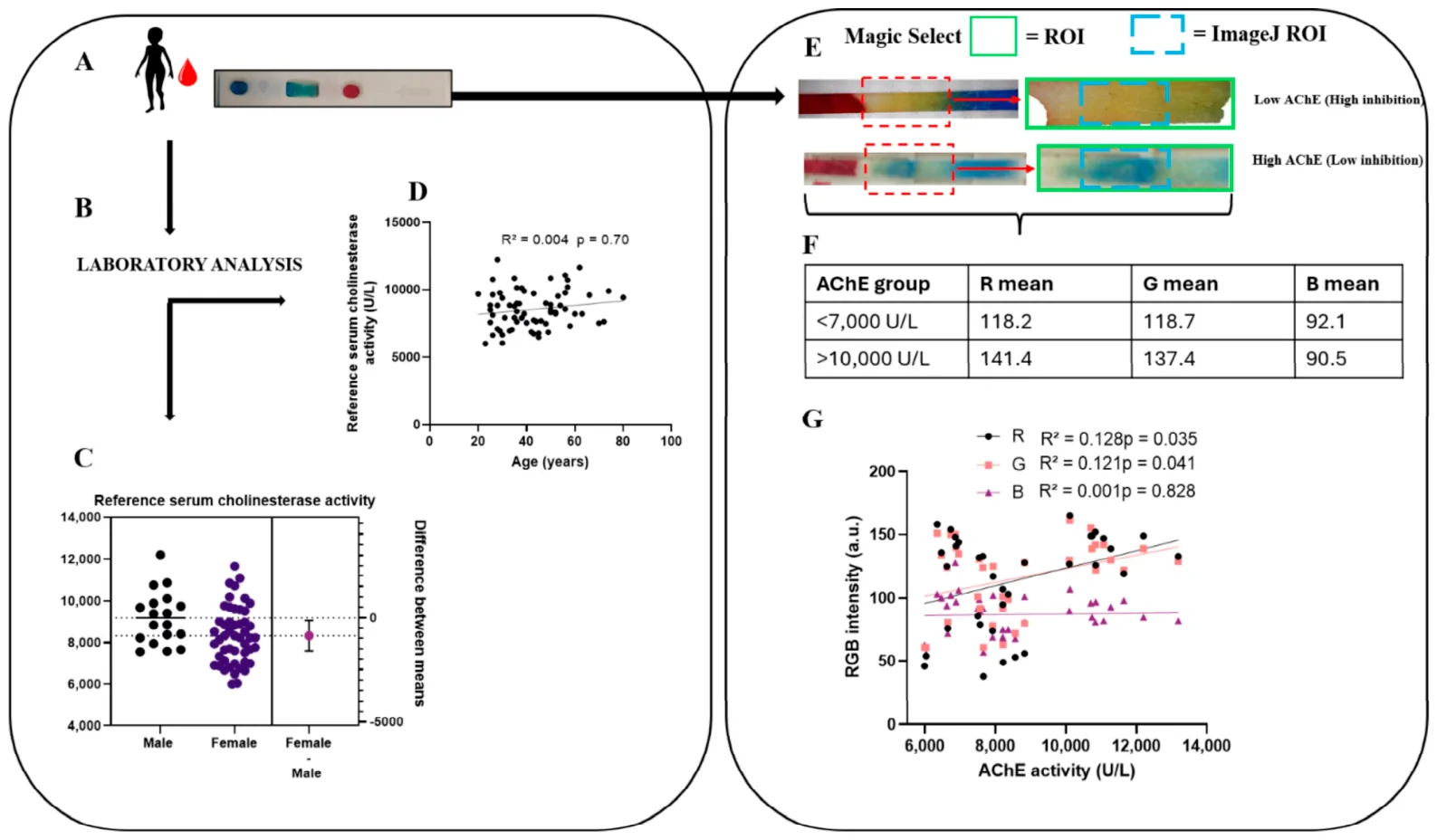
Workflow for the acquisition and analysis of chromatic changes in human capillary blood samples. (**A**) Capillary blood collection from the fingertip and application onto the reagent strip. (**B**) A certified clinical laboratory was used as the reference method for determining serum cholinesterase activity. (**C**) Estimation plot showing AChE activity according to sex. (**D**) Linear regression analysis between age and AChE activity, showing no significant correlation. Panels C and D correspond to the full study population (n = 68). (**E**) Representative colorimetric response along the strip after contact with the blood sample, illustrating the chromatic gradient generated under low and high AChE activity. The dashed red rectangle indicates the reaction area selected for analysis. The green outline represents the ROI identified using the semi-automated Magic Select segmentation tool, and the blue outline indicates the final ROI analyzed in ImageJ for extraction of the mean RGB intensity values. (**F**) Digital image acquisition and analysis of ROI zone and extraction of mean intensity values from the RGB channels. (**G**) Correlation between RGB channel intensity values and AChE activity measured by the reference method. Panels (**E**–**G**) correspond to a subset of samples analyzed for colorimetric response (n = 35).

Reference serum cholinesterase activity was first evaluated across the study population. A statistically significant difference was observed between males and females (Figure 4C; *p* = 0.0223; Supplementary Table S1). However, no significant association was found between age and serum cholinesterase activity, as shown by linear regression analysis (Figure 4D; Supplementary Table S1). Most individuals exhibited preserved basal cholinesterase activity. According to the reference laboratory measurements, samples were classified into three categories based on serum cholinesterase activity: <7000 U/L (n = 11), 7000–10,000 U/L (n = 47), and >10,000 U/L (n = 10). These categories were subsequently used to explore the relationship between chromatic patterns and cholinesterase activity. To further explore the colorimetric response of the test strips, digital image analysis was performed in a subset of samples. Mean intensity values for the RGB channels were obtained from selected regions of interest (ROI) using ImageJ (Figure 4E–G; Supplementary Table S2). When RGB values were compared with the reference serum cholinesterase activity, significant associations were observed for both the R (R^2^ = 0.128, *p* = 0.035) and G (R^2^ = 0.121, *p* = 0.041) channels. In contrast, no significant relationship was found for the B channel (R^2^ = 0.001, *p* = 0.828) (Figure 4G; Supplementary Table S2).

### 3.5. RGB-Based Colorimetric Analysis Under Controlled Experimental Conditions

**Figure 5:**
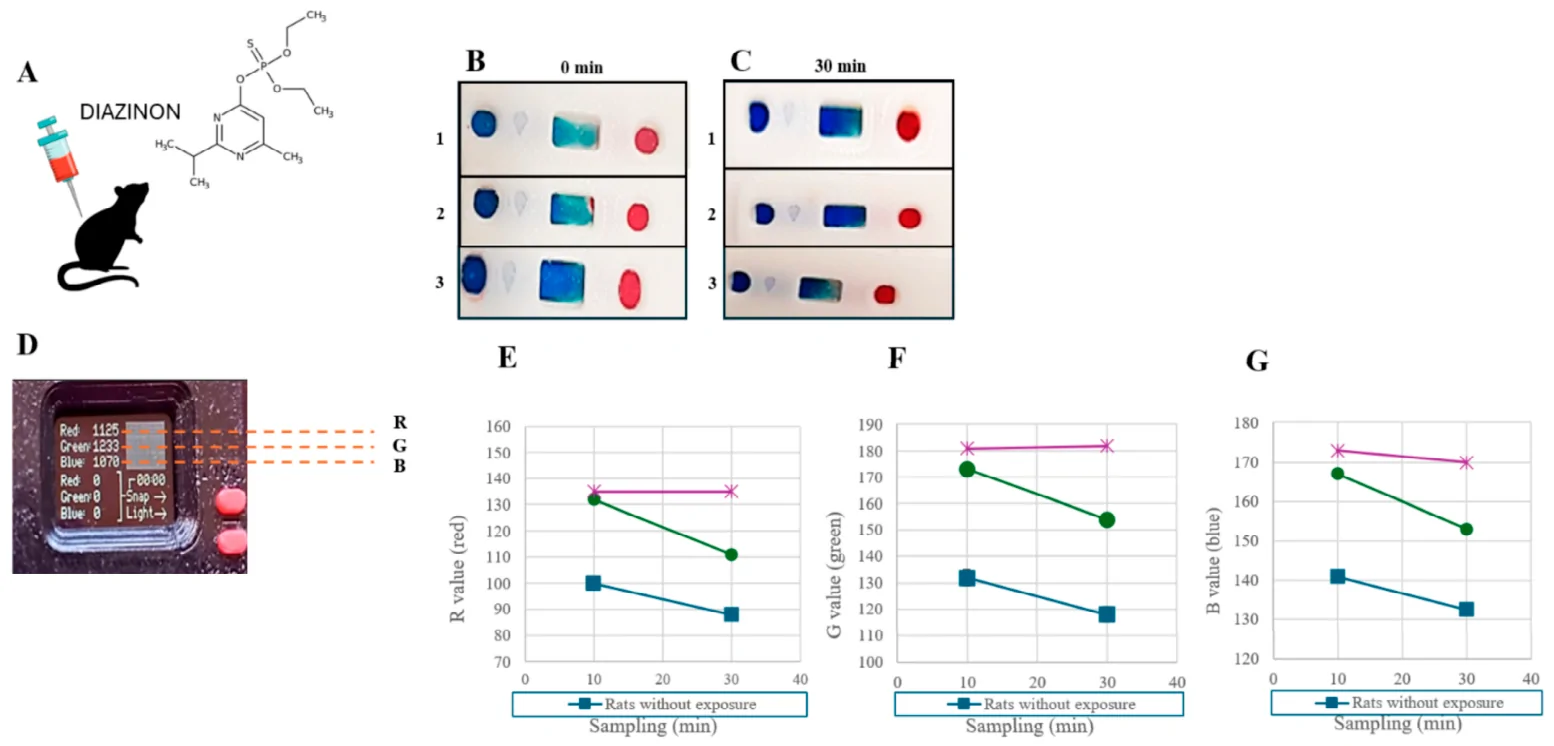
RGB-based colorimetric analysis. (**A**) Representation of the experimental model of exposure to diazinon. (**B**) Reagent strips loaded with capillary blood samples collected immediately before diazinon administration (0 min) and read after 10 min of strip incubation; rows correspond to independent biological replicates (n = 3). (**C**) Reagent strips loaded with capillary blood samples collected from rats 30 min after diazinon administration and read after 10 min of strip incubation; rows correspond to independent biological replicates (n = 3). (**D**) Image of the colorimetric detection device showing the reference color ranges used for visual interpretation of the reaction zone. The displayed values correspond to the intensity of the RGB channels, expressed as numerical outputs generated during color acquisition. These values represent the relative contribution of each color channel within the selected detection area. (**E**–**G**) Quantitative analysis of the RGB color channels obtained from the reaction area: (**E**) red (R), (**F**) green (G), and (**G**) blue (B) channel intensity values.

To further characterize the colorimetric response of the paper-based biosensor under controlled experimental conditions, RGB analysis was performed using the developed colorimetric detection device (Figure 5A–D). Blood samples collected at 0 and 30 min after diazinon administration were applied onto the test strips and incubated for 10 min before analysis. In samples collected at 0 min, the BTB indicator shifted from its original blue color to greenish and yellow tones. In contrast, samples collected 30 min after diazinon administration, in which AChE activity was inhibited or markedly reduced, showed limited substrate reaction; consequently, no proton release occurred and the reaction zone remained dark blue, closely resembling the original color of the BTB indicator. This colorimetric behavior was fully consistent with the results obtained in tube- and plate-based assays, confirming that the paper-based format reliably reproduces the enzymatic response observed under conventional conditions. Analysis of the RGB channels revealed diazinon-dependent color changes between samples collected at 0 and 30 min after diazinon administration (Figure 5E–G; Supplementary Table S3). In samples collected at 0 min, the signal showed a greater relative contribution from the green channel (G ≈ 130) compared with the blue (B ≈ 140) and red (R ≈ 100) channels, resulting in a blue-green hue consistent with the presence of AChE activity. In samples collected 30 min after diazinon administration, a change in the ratio between the color channels was observed, characterized by a relative decrease in the green channel and increased predominance of the blue channel, whereas the red channel remained at lower values. These findings demonstrate that diazinon-induced AChE inhibition can be monitored through quantitative RGB analysis under the experimental conditions evaluated. Since this experiment was designed as a proof-of-concept evaluation, only the baseline (0 min) and post-exposure (30 min) time points were included, as the objective was to compare the absence and presence of AChE inhibition rather than to characterize its temporal progression.

## 4. Discussion

This study demonstrates the feasibility of adapting the Edson method modified by Limperos and Ranta into a portable paper-based platform for the screening of AChE activity. Under controlled experimental conditions, both the conventional tube assay and its microvolume adaptation produced consistent colorimetric responses, characterized by a transition from blue tones corresponding to the initial pH conditions of the assay, to green-yellow hues associated with medium acidification following substrate hydrolysis. This response was observed in samples with preserved enzymatic activity and was absent in diazinon-treated animals, supporting the ability of the assay to detect AChE inhibition. These findings are consistent with the rapid cholinesterase-screening approach originally described by Limperos and Ranta [10]. Importantly, reducing the reaction volume did not compromise the ability of the system to discriminate between preserved and inhibited AChE activity. This miniaturization step was essential for the subsequent implementation of the assay in a paper-based format, in agreement with the concept of microfluidic paper-based analytical devices proposed by Martinez et al. [19]. The present work therefore focuses on the feasibility of translating this classical colorimetric assay into a portable paper-based platform suitable for rapid field screening, rather than on developing a new enzymatic detection principle. The innovation of the present work lies in the integration of this established colorimetric assay into a portable paper-based platform for direct analysis of finger-prick capillary whole blood, combined with RGB-assisted signal evaluation and its preliminary validation under real field conditions through comparison with a certified clinical laboratory.

Unlike classical kinetic assays, this method is not intended to determine enzymatic parameters, but rather to provide a functional readout based on pH-dependent color changes. Accordingly, the assay is particularly suitable for distinguishing between preserved and inhibited AChE activity and for identifying marked enzyme inhibition, although its ability to resolve subtle differences within narrow physiological ranges may be limited. From a device development perspective, the successful miniaturization of the assay into a paper-based format without compromising functionality represents an important step toward point-of-care and field-deployable applications. The combination of capillary-driven fluidics and a colorimetric readout enables the integration of sample handling, enzymatic reaction, and visual detection within a single platform, thereby reducing operational complexity and facilitating rapid analysis.

Evaluation of the different solid supports revealed that strips fabricated using specialized lateral flow papers provided the most consistent performance for the development of a portable rapid-reading system. Although information specifically describing the LF1 substrate is limited, our findings are consistent with previous studies showing that paper selection is a key determinant of capillary flow quality, liquid front uniformity, and colorimetric signal reproducibility. In particular, papers designed for microfluidic applications, including lateral flow membranes and fiberglass-based diagnostic papers, exhibit high porosity, controlled fiber size distribution, and rapid absorption capacity, characteristics that promote stable sample migration and minimize lateral dispersion [16,20]. In agreement with these observations, studies in paper microfluidics have demonstrated that pore size, fiber structure, and paper density influence capillary front stability and transport uniformity, ultimately affecting the reproducibility of the colorimetric signal [13,14]. In our prototype, the use of a support with optimized capillary flow properties generated a more defined and consistent chromatic signal, suggesting improved reagent–sample interactions during flow. These characteristics are particularly relevant for ensuring reliable performance under variable field conditions, where fluid handling and signal stability are critical. Additionally, maintaining the initial pH conditions of the assay keeps the acid–base indicator in a stable baseline state in its deprotonated form, providing a stable, high-contrast baseline. Under these conditions, the absence of a color shift reflects a lack of AChE-mediated substrate hydrolysis rather than an effect of the basic environment itself, thereby reducing background noise and facilitating visual interpretation, which contributes to the overall robustness of the system [21]. However, because the colorimetric signal may be influenced by factors such as reaction time, sample volume, and image acquisition conditions, future implementation of this platform may benefit from the incorporation of internal references or correction procedures to reduce inter-test variability and improve result comparability. Likewise, variations in illumination and image capture devices may affect color perception, highlighting the importance of standardized acquisition conditions for reliable digital analysis under field conditions.

The absence of differences between AChI and AChClO_4_ can be explained by the fact that the acetylcholine cation, rather than the accompanying anion, is the functionally relevant moiety recognized by AChE. Through its catalytic triad (Ser-His-Glu), AChE hydrolyzes acetylcholine to produce choline and acetic acid [22,23]. Consequently, perchlorate and iodide mainly contribute to salt solubility and stability without directly affecting catalysis, which is consistent with classical AChE assays in which different choline ester salts exhibit comparable hydrolysis properties [11]. During assay standardization, both substrates generated similar colorimetric responses; however, AChClO_4_ produced a more easily distinguishable visual color transition and was therefore selected for subsequent experiments. The use of serum, which presents lower optical interference than whole blood, may have further contributed to minimizing variability associated with the counterion. These findings support the robustness of the pH-based detection approach using different acetylcholine salts. Under controlled experimental conditions, RGB analysis enabled consistent discrimination between preserved AChE activity and enzyme inhibition over time. Samples collected 0 min after diazinon administration exhibited a relative predominance of the green channel, consistent with the pH-dependent color shift associated with substrate hydrolysis. In contrast, samples collected 30 min after diazinon administration showed increased predominance of the blue channel, reflecting attenuation of the pH change induced by AChE inhibition. These chromatic changes were consistently observed among the analyzed samples, suggesting that RGB analysis may provide an objective approach for detecting variations in enzyme activity under controlled experimental conditions. This behavior is consistent with the classical principles of AChE activity and OP inhibition, in which acetylcholine hydrolysis generates acidic products that induce a local decrease in pH, a phenomenon historically exploited in colorimetric assays such as the Ellman method [11]. Likewise, the predominance of the blue channel observed after diazinon exposure agrees with the well-established mechanism by which OPs form stable complexes with the active site of AChE and block its catalytic function [24]. The ability of RGB analysis to detect these chromatic changes is consistent with previous studies demonstrating the utility of paper-based colorimetric platforms and digital image analysis for monitoring enzymatic reactions and pH variations [19,25]. Taken together, these findings suggest that RGB-based analysis represents a feasible approach for evaluating AChE activity and inhibition under controlled experimental conditions, supporting its potential application as a semi-quantitative analytical strategy. In addition, RGB-assisted digital analysis provides an accessible approach for signal evaluation and may facilitate the future implementation of portable imaging systems and field-based workflows.

The application of RGB analysis to human blood samples showed that the colorimetric platform can be used under real field conditions. Although this study was not intended to establish diagnostic thresholds, the results support the use of the proposed device as a preliminary screening tool for AChE activity. Further studies with larger populations will be needed to optimize the method and define its diagnostic performance.

When RGB-derived signals were compared with the reference serum cholinesterase activity measured in the clinical laboratory, lower enzymatic activity was associated with darker color tones, whereas higher activity corresponded to lighter tones. Importantly, this response was mainly driven by changes in the R and G channels, while the B channel showed no significant association. These findings are consistent with previous studies by Kostelnik et al. [14] and Pohanka and Zakova [17], who demonstrated the applicability of RGB-based image analysis for cholinesterase assays through validation against the standard Ellman’s method. Together, these studies support the use of digital image analysis as an objective approach for assessing cholinesterase activity. This pattern is consistent with the pH-dependent mechanism of the assay, in which increased enzymatic activity promotes substrate hydrolysis and acidification of the reaction medium.

Although the linear correlations between RGB values and AChE activity were relatively weak (R^2^ < 0.2), the method demonstrated the ability to discriminate between different activity ranges. Participants grouped as <7000 U/L and >10,000 U/L exhibited distinct chromatic profiles, mainly associated with differences in the red and green channels, suggesting that RGB analysis may complement the visual assessment of different enzyme activity ranges rather than provide precise numerical predictions. Similar chromatic trends have been described in experimental models; however, human samples exhibited greater variability, likely reflecting the increased biological and environmental complexity of capillary blood, including hematocrit-dependent variability, hemoglobin-related optical interference, and interindividual differences that may attenuate the colorimetric signal [26]. Accordingly, the observed R^2^ values should be interpreted as describing the strength of the linear association between RGB values and AChE activity, rather than as the sole indicator of the analytical performance of the proposed method. As discussed by Lemyre et al. [27] and Sánchez et al. [28], the coefficient of determination is influenced by data variability and should not be used in isolation as evidence of model linearity or analytical performance. Despite these limitations, the overall agreement between chromatic classification and AChE values suggests that RGB analysis may be useful as a complementary semi-quantitative approach for rapid stratification of enzyme activity in human samples. Accordingly, the proposed platform is not intended to replace conventional analytical techniques, but rather to serve as a preliminary screening and monitoring strategy, particularly in field or low-resource settings where access to specialized instrumentation is limited. Taken together, these findings support the potential translation of laboratory-based assays into practical on-site monitoring tools, facilitating rapid decision-making in occupational and environmental health settings. An additional strength of the present study is that the RGB-based colorimetric response was compared with cholinesterase activity measured by an independent clinical laboratory, providing an external reference for evaluating the relationship between the colorimetric response and enzyme activity under real-world conditions.

Although the device is capable of distinguishing between preserved and inhibited AChE activity, further optimization will be required to improve signal consistency, particularly when analyzing complex biological matrices such as human capillary blood. In addition, environmental conditions and image acquisition variability may affect the reproducibility of RGB measurements. Therefore, future studies should focus on improving signal standardization, optimizing device design, refining digital analysis, and validating performance in larger and more diverse populations. Recent studies have shown that the integration of artificial intelligence with paper-based colorimetric biosensors can improve the robustness and quantitative interpretation of colorimetric signals, highlighting the potential of computational image analysis to further enhance portable sensing platforms [29]. Although more sophisticated optical biosensing approaches have been reported, simple colorimetric assays based on established enzymatic reactions remain attractive because of their low cost, ease of use, and suitability for rapid screening in point-of-care and field settings. Similarly, paper-based AChE biosensors integrated with portable readers and smartphone applications have recently emerged as promising platforms for on-site organophosphate detection, further supporting the development of portable point-of-care technologies [30]. In addition, the present study represents a proof-of-concept evaluation of the proposed platform. Therefore, comprehensive analytical validation, including the determination of sensitivity, specificity, limit of detection, limit of quantification, reproducibility, storage stability, and robustness under variable field conditions, will be addressed in subsequent stages of device development. Future studies will also evaluate alternative color-space models and more advanced image analysis approaches to further improve the quantitative performance of the proposed platform.

Despite these limitations, taken together, these findings support the potential utility of the proposed colorimetric platform as a simple, rapid, and accessible tool for the preliminary screening of AChE activity. The implementation of the assay in a paper-based format, together with its compatibility with RGB-assisted digital analysis and its evaluation in human samples through comparison with cholinesterase activity measured in a clinical laboratory, provides preliminary evidence supporting its potential application for monitoring OP exposure in field and low-resource settings.

## 5. Conclusions

The pH-based colorimetric assay enabled reproducible detection of AChE activity and its inhibition across different biological matrices. Miniaturization of the reaction allowed its successful implementation into a paper-based format while preserving its ability to discriminate between preserved and inhibited AChE activity. In combination with paper substrates exhibiting controlled capillary flow properties, the platform generated a semi-quantitative response that could be further characterized by RGB-assisted digital analysis. Taken together, these findings demonstrate the feasibility of the proposed platform as a preliminary proof-of-concept for visual and RGB-assisted screening of AChE activity. The successful translation of the assay into a miniaturized format, together with its compatibility with digital image analysis and evaluation in human samples, highlights its potential utility for point-of-care and low-resource applications where access to conventional laboratory infrastructure is limited.

## 6. Patents

The authors have a patent application and/or registration related to this work.

## Data Availability

The data supporting the findings of this study are available within the article and its Supplementary Materials. Additional data are available from the corresponding author upon reasonable request.

## Supplementary Materials

The following supporting information is available online at https://www.mdpi.com/article/10.3390/bios16090490/s1: Table S1. Demographic characteristics and serum cholinesterase activity of the study population; Table S2. RGB colorimetric analysis of test strips from human participants; Table S3. Raw RGB intensity values obtained from colorimetric analysis of reaction strips in rat blood samples under different experimental conditions; Figure S1. Optical acquisition systems used in this study.

## Abbreviations

AChClO_4_	Acetylcholine perchlorate
AChE	Acetylcholinesterase
AChI	Acetylcholine iodide
BTB	Bromothymol blue
LED	Light-emitting diode
LF1	Whatman LF1 separator paper
OP	Organophosphate
RGB	Red, Green, Blue
ROI	Region of interest
SD	Standard deviation
