# Supplementary material for: Portable Paper-Based Colorimetric Biosensor for Rapid Screening of Organophosphate Exposure via Acetylcholinesterase Activity and RGB Image Analysis
Source: Biosensors (Basel). 2026 Sep 3;16(9):490. doi: 10.3390/bios16090490 (PMC13604447; doi:10.3390/bios16090490)
Supplement: Supplementary file 1 [file biosensors-16-00490-s001.zip › biosensors-4435218-supplementary.pdf]

Portable Paper-Based Colorimetric Biosensor for Rapid Screening of Organophosphate Exposure via Acetylcholinesterase Activity and RGB Image Analysis

Supplementary Figure S1.

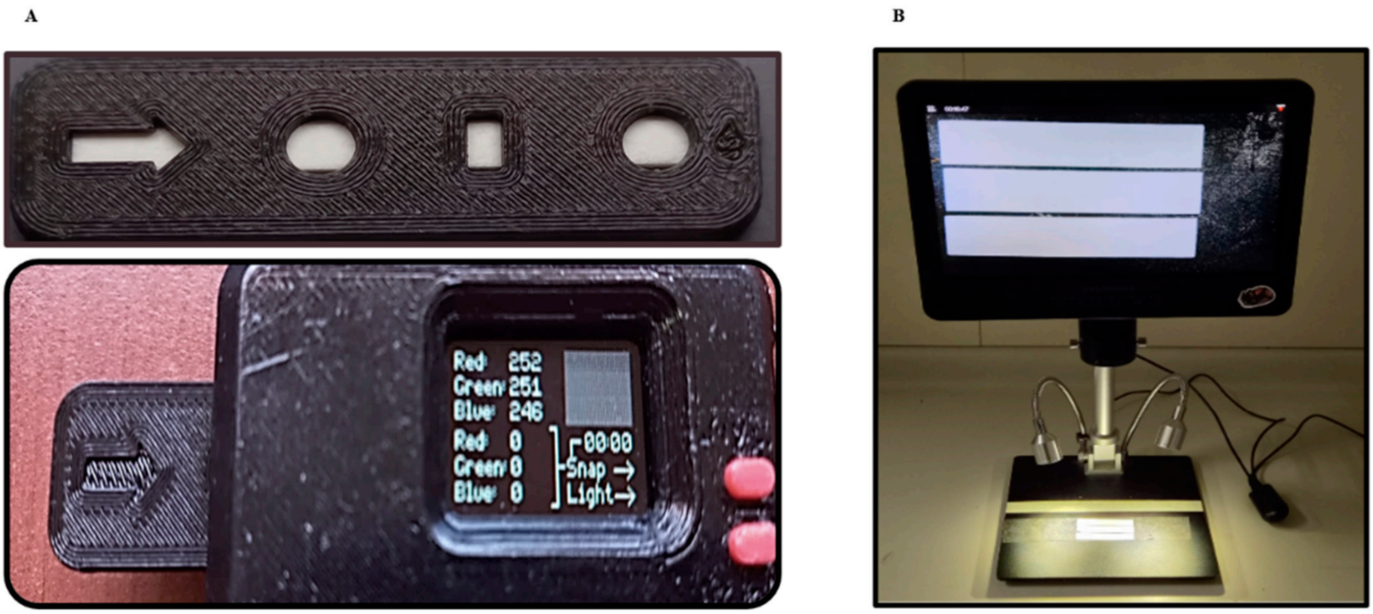

Optical acquisition systems used in this study. (A) Portable RGB reader showing the custom-designed 3D-printed strip holder used for image acquisition. (B) Fixed camera support used for ImageJ image acquisition under controlled illumination.

Supplementary Table S1 Demographic characteristics and serum cholinesterase activity of the study population

Code	Sex	Age (years)	Cholinesterase Activity (U/L)	Cholinesterase Activity (%)	Occupation	Exposure to OP within the previous 15 days
CB19D2343	F	26	6630	84	Healthcare	No
CB19D2346	F	29	6965	85	Healthcare	No
CB14	F	28	7115	85	Healthcare	No
CB19D2351	F	25	7598	87	Healthcare	No
CB19D2335	F	26	8134	89	Healthcare	No
CB19D2342	F	25	8524	90	Healthcare	No
CB19D2338	M	25	8853	87	Healthcare	No
CB12	M	20	9680	89	Education	No
CB19D2349	M	26	10752	92	Healthcare	No
CB19D2350	M	28	12210	98	Healthcare	No
CB20	F	33	6971	85	Unemployed	No
CB19D2345	F	34	7052	85	Healthcare	No
CB15	F	36	7646	87	Unemployed	No
CB19D2330	F	31	7932	88	Household	No
CB19D2354	M	36	8400	85	Healthcare	No
CB19D2347	F	35	8782	91	Healthcare	No
CB19D2333	F	36	8992	92	Healthcare	No
CB19D2341	M	30	9384	88	Healthcare	No

CB19D2353	M	39	9889	90	Healthcare	No
CB24	M	38	10108	91	Healthcare	No
CB19D2325	F	36	10123	96	Education	No
CB08	F	35	10845	99	Healthcare	No
CB23	F	45	6474	79	Healthcare	No
CB19	F	43	6741	80	Healthcare	No
CB25	F	45	6789	80	Healthcare	No
CB19D2327	F	49	6869	80	Agriculture	No
CB17	F	42	6891	80	Healthcare	No
CB18	F	46	7705	83	Healthcare	No
CB19D2352	F	43	7758	83	Healthcare	No
CB19D2336	F	48	9004	87	Healthcare	No
CB19D2337	F	43	9707	89	Healthcare	No
CB02	F	58	7328	82	Healthcare	No
CB19D2348	F	52	8226	85	Healthcare	No
CB19D2339	F	50	8321	85	Healthcare	No
CB19D2344	F	52	8321	85	Healthcare	No
CB04	F	50	8520	86	Arts	No
CB19D2331	F	53	9521	89	Healthcare	No
CB19D2332	F	56	9765	90	Healthcare	No
CB19D2340	F	57	10183	91	Healthcare	No
CB19D2324	F	57	10717	93	Unemployed	No
CB21	F	56	11079	94	Healthcare	No
SR01	F	26	9625	94	Agriculture	No
SR08	F	23	5998	81	Agriculture	No
SR031	F	28	8833	91	Unemployed	No
SR025	M	29	9749	90	Education	No
SR012	F	33	8853	91	Healthcare	No
SR014	F	38	7926	88	Household	No
SR017	F	30	6655	84	Healthcare	No
SR018	F	30	6040	81	Healthcare	No
SR021	F	39	8226	89	Household	No
SR032	F	37	8979	92	Unemployed	No
SR027	M	35	7940	84	Healthcare	No
SR016	F	48	7506	82	Healthcare	No
SR029	F	44	7667	83	Healthcare	No
SR011	M	40	7572	82	Unemployed	No
SR022	F	56	8568	86	Agriculture	No
SR030	F	50	8890	87	Healthcare	No
SR013	M	50	8371	85	Unemployed	No
SR015	M	50	10854	93	Unemployed	No
SR05	M	54	8833	87	Agriculture	No
SR04	F	66	9591	89	Household	No
SR09	F	60	8218	84	Unemployed	No
SR028	F	62	11654	96	Unemployed	No
SR010	M	63	8218	84	Agriculture	No
SR020	F	74	9890	90	Household	No
SR019	M	70	7548	82	Retired	No
SR02	M	72	7651	83	Household	No
SR024	M	80	9413	88	Household	No

**Characteristic**	**Overall (n = 68)**	**Female (n = 50)**	**Male (n = 18)**

**Age (years), mean ± SD**	43.0 ± 13.9	42.8 ± 12.4	43.6 ± 17.9
**Cholinesterase Activity (U/L), mean ± SD**	8555.5 ± 1386.1	8327 ± 1361	9190 ± 1284
**Cholinesterase Activity (%), mean ± SD**	87.2 ± 4.6	87.0 ± 4.7	87.7 ± 4.2
**Residence, Rural, n (%)**	68 (100)	50 (100)	18 (100)
**Occupation, n (%)**			
Healthcare	40 (58.8)	32 (64.0)	8 (44.4)
Agriculture	6 (8.8)	4 (8.0)	2 (11.1)
Education	3 (4.4)	1 (2.0)	2 (11.1)
Household	7 (10.3)	5 (10.0)	2 (11.1)
Unemployed	10 (14.7)	7 (14.0)	3 (16.7)
Arts	1 (1.5)	1 (2.0)	0 (0.0)
Retired	1 (1.5)	0 (0.0)	1 (5.6)
**Exposure to OP within the previous 15 days, n (%)**			
Yes	-	-	-
No	68 (100)	50 (100)	18 (100)

Demographic, occupational, and biochemical characteristics of the study population. Serum cholinesterase activity values (U/L) were determined by a certified clinical laboratory (Laboratory Loncomilla). The table summarizes age, serum cholinesterase activity (U/L), relative serum cholinesterase activity (%), sex distribution, occupation, place of residence, and self-reported exposure to organophosphate pesticides within the previous 15 days. All participants were residents of rural areas. Continuous variables are presented as mean ± standard deviation (SD), and categorical variables are presented as n (%).

Supplementary Table S2

Code	Cholinesterase Activity (U/L)	R value	G value	B value
SR08	5998	46	61	63
SR018	6040	54	61	56
cb01	6354	158	151	103
cb23	6474	136	134	100
CB19D2343	6630	125	124	94
SR017	6655	76	81	72
cb19	6741	154	150	102
CB19D2327	6869	148	150	128
cb17	6891	141	140	97
CB19D2346	6965	144	135	106

SR016	7506	86	101	92
SR019	7548	132	131	99
SR011	7572	79	92	90
SR02	7651	133	124	92
SR029	7667	38	61	57
SR014	7926	74	78	69
SR027	7940	117	125	102
SR09	8218	107	101	75
SR010	8218	95	92	69
SR021	8226	49	63	68
SR013	8371	103	99	75
SR022	8568	53	72	68
SR031	8833	56	80	81
SR05	8833	128	128	101
CB19D2325	10123	165	161	107
Cb24	10108	127	130	90
CB19D2324	10717	149	155	96
CB19D2349	10752	149	139	85
SR015	10854	126	122	97
cb08	10845	152	142	81
cb21	11079	147	142	82
cb07	11285	139	130	93
SR028	11654	119	122	98
CB19D2350	12210	149	139	85
cb10	13200	133	129	82

RGB colorimetric analysis of test strips. RGB values (red, green, and blue channels) were obtained through digital image analysis using ImageJ software. Data correspond to a subset of participants included in Fig 4, for whom image-based colorimetric analysis was performed. RGB values represent the mean intensity extracted from a defined region of interest (ROI) on each test strip.

Supplementary Table S3

RATS	TIME E	TIME C [min]	R	G	B
R1 662 gr 12.5 ul	T0	10	100	135	141
	T0	10	136	175	160
	T1	10	109	151	160
	T1	30	130	171	162
	T2	10	176	190	173
	T2	30	183	200	168
R2 616 gr 25ul	T0	10	98	131	141
	T0	10	119	153	143
	T1	10	102	143	166
	T1	30	109	149	165
	T2	10	165	200	185
	T2	30	177	213	190
R3 612 gr 50ul	T0	10	97	125	146
	TO	10	100	132	141
	T1	10	132	173	167
	T1	30	111	154	153
	T2	10	135	181	173
	T2	30	135	182	170

Raw RGB intensity values obtained from colorimetric analysis of reaction strips in rat blood samples under different experimental conditions. RGB values correspond to the mean intensity of the red (R), green (G), and blue (B) channels extracted from the selected region of interest (ROI) using ImageJ software. Measurements were performed at different experimental time points (T0, T1, T2) and incubation times (10 and 30 min), under distinct experimental conditions (A: control; B: treated). Each dataset corresponds to individual animals (R1–R3) with their respective body weights and sample volumes. Variations in RGB values reflect changes in chromatic response associated with AChE activity and its modulation under the evaluated conditions.
